# Tailored translation: Local tRNA activation to match protein composition needs

**DOI:** 10.1093/plcell/koaf204

**Published:** 2025-08-20

**Authors:** Laura Arribas-Hernández

**Affiliations:** Assistant Features Editor, The Plant Cell, American Society of Plant Biologists; Instituto de Hortofruticultura Subtropical y Mediterránea La Mayora (IHSM), Consejo Superior de Investigaciones Científicas—Universidad de Málaga (CSIC-UMA) . Bulevar Louis Pasteur, 49, Málaga 29010, España

Both DNA and proteins are formed by a succession of chemical elements that, like the letters inside a sentence, are arranged in sequences. But because they are written in different languages, translation requires the action of a “molecular dictionary”: the cellular repertoire of transfer RNAs (tRNAs) capable of decrypting the genetic code.

tRNAs reside in the genome as tRNA genes (tDNAs) that, for reasons not yet well understood, appear in widely variable copy numbers dispersed along chromosomal arms in most eukaryotes, sometimes grouped in clusters whose distribution is highly lineage-specific (Bermudez-Santana et al. 2010). In the flowering plant Arabidopsis (*A. thaliana*), many copies of serine (S) and tyrosine (Y) tDNAs form a distinct “SYY cluster” in chromosome 1 (Beier et al. 1991). However, this region appears to be epigenetically silenced in seedlings (Hummel et al. 2020), raising the question of whether these tDNA copies are functional. In new work, **Guillaume Hummel and colleagues (Hummel et al. 2025)** found that the transcriptional activation of the SYY cluster occurs in a cell-type-specific manner and is required for the efficient translation and stability of Ser/Tyr/Pro-rich proteins produced in these cells.

To determine whether expression of the SYY cluster is restricted to specific tissues and/or developmental stages, Hummel et al. dissected and analyzed germinating and young seedlings by RNA blot, using a probe specific for tRNAs^Tyr^ derived from the SYY cluster and not from dispersed copies. These analyses unequivocally detected SYY cluster-derived tRNAs in root tips, and whole mount in situ hybridization revealed a gradual increase in abundance along the root axis toward the meristematic zone, with a maximum in the peripheral columella and adjacent lateral root cap cells (Fig.) (Hummel et al. 2025). Interestingly, these tissues are constantly being renewed: as the root grows, the inner layer divides while the outer layer is shed, maintaining a steady organ size. In some plant species, the discarded cells become long-lived “border cells” that secrete a mucilage rich in hydroxyproline-rich glycoproteins, which protects the tip and is crucial for root–microbe interactions.

**Figure.**
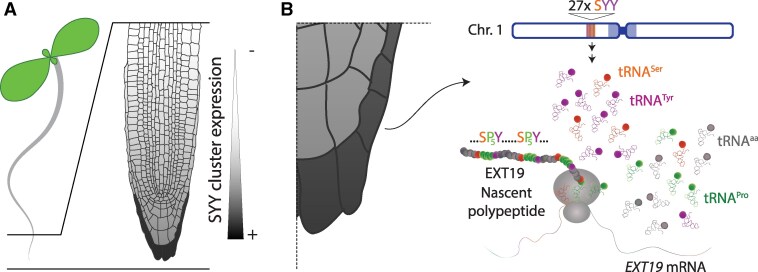
Activation of the Arabidopsis tRNA SYY cluster in cells of the root cap fuels translation of Ser/Tyr/Pro-rich proteins. **A)** Graphic representation of the restricted expression of the SYY cluster in Arabidopsis seedlings. **B)** EXT19 contains a large number of Ser-Pro_5_-Tyr repeats, whose translation in root cap cells is fueled by the extra supply of tRNA^Ser/Tyr^ resulting from the transcriptional activation of the SYY cluster (27xSYY) in chromosome 1. The root tip graphs are from Erin Sparks, available on FigShare under a CC BY 4.0 license (https://figshare.com/articles/figure/Arabidopsis_root_anatomy/4688344). Figure credit: L. Arribas-Hernández.

Hummel et al. observed that SYY cluster-derived tRNAs are not essential for the development of the root cap, because CRISPR/Cas9-generated mutants devoid of the entire SYY cluster, *syy-1* and *syy-2*, do not display abnormalities in cell morphology or shedding patterns (Hummel et al. 2025). This could indicate that the SYY cluster is involved in a process happening outside the plant and, accordingly, the authors noticed that secreted hydroxyproline-rich glycoproteins of the EXTENSIN (EXT) family are highly rich in Ser and Tyr. Using a fluorescent EXT19 reporter crossed to the *syy-1* knockout line, Hummel et al. elegantly showed that root cap–specific expression of the SYY cluster contributes to the efficient translation and stability of this protein (Fig.) (Hummel et al. 2025).

The study by Hummel et al. demonstrates that the differential expression of a tDNA cluster across plant tissues is key to fueling the translation of cell type–specific proteins with specific amino acid and motif compositions that may be challenging for the ribosome. Comparable results have been obtained for silk-protein constituents produced by *Bombyx mori* caterpillars (Chevallier and Garel 1982), whose silk gland–specific tRNA^Ala^ genes are also clustered within the genome (Underwood et al. 1988), a beautiful example of convergent molecular evolution.

Perhaps the most striking and yet unexplained result from the study by Hummel et al. is that SYY cluster activation can bypass epigenetic silencing, because methylation of this large locus remains unaltered in the root cap cells where SYY cluster transcription occurs (Hummel et al. 2025). The authors discussed whether the subnucleosomal size of tRNA genes (∼150 bp) may enhance RNAPIII transcription by creating nucleosome-free environments. This possibility will certainly be addressed in future work from the group.

## Recent related articles in *The Plant Cell*

- Zhang et al. (2025) described how serine deficiency triggers tRNA^Ser^ degradation and reduced translation elongation at serine codons in maize.
- Yang et al. (2023) reviewed the current knowledge on DNA-dependent RNA polymerases, with an RNAPIII-dedicated chapter that updates our understanding of tRNA transcription initiation in plants.
- In another review article, Wu et al. (2024) recapitulate how tRNAs and tRNA-derived fragments regulate translation.

## Data Availability

No new data were generated or analysed in support of this research.
